# Recent Advances in Understanding the Reversal of Gene Silencing During X Chromosome Reactivation

**DOI:** 10.3389/fcell.2019.00169

**Published:** 2019-09-03

**Authors:** Irene Talon, Adrian Janiszewski, Joel Chappell, Lotte Vanheer, Vincent Pasque

**Affiliations:** Department of Development and Regeneration, Leuven Stem Cell Institute, KU Leuven, Leuven, Belgium

**Keywords:** pluripotency, stem cells, epigenetic memory, X chromosome reactivation, X chromosome inactivation, gene silencing, chromatin, epigenetics

## Abstract

Dosage compensation between XX female and XY male cells is achieved by a process known as X chromosome inactivation (XCI) in mammals. XCI is initiated early during development in female cells and is subsequently stably maintained in most somatic cells. Despite its stability, the robust transcriptional silencing of XCI is reversible, in the embryo and also in a number of reprogramming settings. Although XCI has been intensively studied, the dynamics, factors, and mechanisms of X chromosome reactivation (XCR) remain largely unknown. In this review, we discuss how new sequencing technologies and reprogramming approaches have enabled recent advances that revealed the timing of transcriptional activation during XCR. We also discuss the factors and chromatin features that might be important to understand the dynamics and mechanisms of the erasure of transcriptional gene silencing on the inactive X chromosome (Xi).

## Introduction

X chromosome inactivation (XCI) is a process that evolved in mammals to balance gene expression between XX female and XY male cells (Payer and Lee, 2008; Galupa and Heard, 2018). Early in development, one of the two X chromosomes in female cells is randomly chosen for inactivation. In the mouse and human systems, each parental chromosome is known to have a similar chance of being inactivated. Therefore, XCI is considered random. As a result, females are mosaics with respect to allelic gene expression from the X chromosomes. Moreover, a small subset of genes, termed ‘escapee genes,’ can also escape inactivation and remain active in a tissue and cell type-specific manner (Tukiainen et al., 2017). Because X chromosomes can carry mutated alleles, XCI has important implications for the manifestation of several human diseases. Such diseases can have serious negative health consequences. For example, thousands of young girls have Rett Syndrome, a neurodevelopmental disorder caused by a mutation on the X-linked gene *MECP2* (Bianchi et al., 2012; Lee and Bartolomei, 2013; Carrette et al., 2017; Gribnau and Barakat, 2017). While mutations on the only X-linked gene copy in males can be lethal, the same mutation in females can lead to variable phenotypes. XCI can also be skewed toward one or the other parental chromosome, meaning that one of the X chromosomes will be preferentially silenced. Skewed XCI is observed in some females and might be caused either by negative selection, when a variant present on one of the two X chromosomes is associated with lethality, or may be purely stochastic in nature (Wu et al., 2014; Shvetsova et al., 2019). Today, clinicians still lack effective tools to assess how the severity of a subset of X-linked human disorders is affected by specific patterns of XCI. Hence, it is important to better understand XCI with the goal of improving the prediction, detection, diagnosis, and treatment of several X-linked human disorders.

XCI leads to the coordinated silencing of most genes on the Xi and the formation of stably silenced chromatin via epigenetic processes. The latter refers to changes in gene expression that are inherited from one cell division to another without changes in DNA sequence. After inactivation, the Xi adopts an epigenetic memory of gene silencing, usually stably maintained across cellular divisions. Nevertheless, such chromosome-wide epigenetic memory of XCI is fully reversible in a process known as X chromosome reactivation (XCR) (Mak et al., 2004; Okamoto et al., 2004). During mouse development, XCR occurs in the inner cell mass (ICM) (Mak et al., 2004; Borensztein et al., 2017a) and in primordial germ cells (PGCs) (Sugimoto and Abe, 2007). During human development, XCR takes place in PGCs (Von Meyenn and Reik, 2015), but not in the ICM where both X chromosomes are found active in female cells (Okamoto et al., 2011; Petropoulos et al., 2016). During XCR, many repressive chromatin mechanisms present on the Xi are erased. Studies focusing on XCR have provided valuable insights on how such repressive chromatin mechanisms are erased, on epigenetic reprogramming, the stability of gene silencing and chromatin organization. Understanding XCR could not only provide key insights into gene regulation, but it may also help to design targeted strategies for reactivation of wild-type alleles on the Xi and even on other chromosomes as well. This could be used, for example, to treat Rett Syndrome where it has been shown, in mice, that reactivation of the inactive wild-type *Mecp2* allele in female cells can be beneficial (Guy et al., 2007; Przanowski et al., 2018).

**FIGURE 1:**
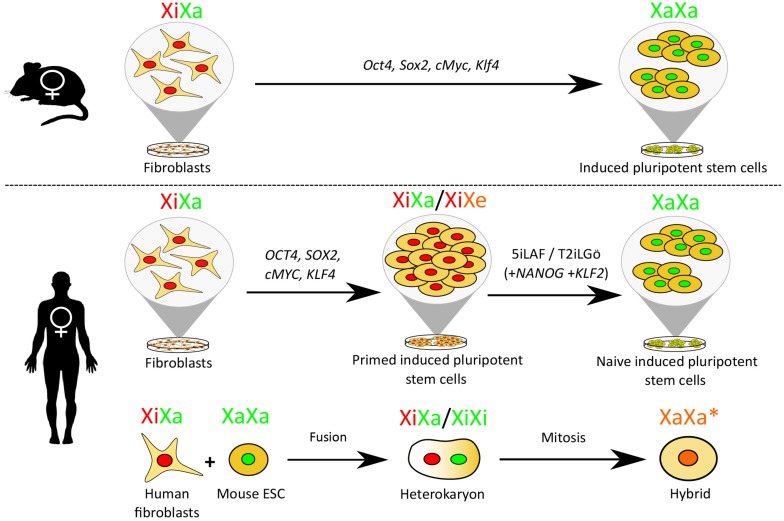
XCR during female mouse and human iPSC reprogramming and following cell fusion. Mouse fibroblasts induced to a pluripotent state by the upregulation of *Oct4, Sox2, cMyc*, and *Klf4* activate their Xi. Human fibroblasts converted to the primed pluripotent state by upregulating OCT4, SOX2, cMYC, and KLF4 do not undergo XCR, further reprogramming to the naïve state using a naïve conversion media [e.g., 5iLAF (which contains inhibitors that target the glycogen synthase kinase-3 (GSK3b), the mitogen-activated protein kinase (MEK), the Rho-associated, coiled-coil containing protein kinase (ROCK), the serine/threonine-protein kinase B-Raf (BRAF) and the proto-oncogene tyrosine-protein kinases (SRC), in addition to human leukemia inhibitor factor (LIF), activin A and fibroblast growth factor (FGF) (Theunissen et al., 2014)] or T2iLGö [which requires overexpression of KLF2 and NANOG in the presence of MEK, GSK-3, protein kinase C (PKC), ROCK inhibitor and human LIF (Takashima et al., 2014)] is required to induce XCR. Partial human XCR is also achievable through cell fusion, whereby a human fibroblast is fused to a mouse embryonic stem cell (mESC). Following mitosis, a hybrid cell is formed containing chromosomes from both mouse and human in which the human X chromosome is partially reactivated. Red nuclei = XiXa state, green nuclei = XaXa state, XaXa^∗^ = partially reactivated X, XiXe = eroded X.

To study XCR, several experimental *in vitro* procedures have been used (Figure 1). These include nuclear transfer (Eggan et al., 2000; Pasque et al., 2011), reprogramming somatic cells into induced pluripotent stem cells (iPSCs) (Maherali et al., 2007), conversion of primed human pluripotent stem cells (hPSCs) to a naïve-like state of pluripotency (Theunissen et al., 2016; Sahakyan et al., 2017; Vallot et al., 2017) and cell fusion-mediated pluripotent reprogramming (Cantone et al., 2016). Partial XCR may affect one or more genes and the level of gene expression may or may not reach the corresponding level on the active X chromosome (Xa). Complete XCR leads to full erasure of epigenetic memory of XCI. This is best seen in female mouse iPSCs originating from female mouse fibroblasts with an Xi, where iPSC differentiation induces random XCI (Maherali et al., 2007). Therefore, XCR provides a remarkable example to study chromosome-wide erasure of gene silencing and reversal of epigenetic memory.

How is gene silencing reversed during XCR? Transcription factors (TFs) are good candidate inducers of epigenetic memory erasure, but their precise role during XCR remains unclear. For XCR to take place, the expression of the long non-coding RNA (lncRNA) *Xist* has to be repressed. In addition, multiple chromatin modifications that synergistically maintain the silenced state of the Xi in somatic cells need to be erased (Csankovszki et al., 2001). The enrichment of chromatin marks associated with gene repression such as H3K27me3, macroH2A as well as DNA methylation, have been shown to be reversed during XCR (Mak et al., 2004; Okamoto et al., 2004; Maherali et al., 2007; Pasque et al., 2014; Borensztein et al., 2017a). The dynamics of XCR in the ICM and during reprogramming to iPSCs have been well-defined by immunofluorescence and RNA fluorescence *in situ* hybridization (RNA-FISH) analyses (Pasque et al., 2014; Borensztein et al., 2017a). However, how *Xist* silencing is linked to chromosome-wide erasure of transcriptional silencing remains unclear.

Recent advances in sequencing have enabled researchers to define the precise timing of X-linked gene reactivation for many genes in multiple experimental systems. While several mechanistic insights have been obtained, the precise factors and mechanisms that underlie the coordinated reactivation of silenced genes on the Xi during XCR remain to be defined. Here, we identify and discuss new studies focusing on the dynamics of XCR, as well as the factors and possible mechanisms underlying reversal of gene silencing.

## X Inactivation and Reactivation in Mouse Development and Reprogramming

**FIGURE 2:**
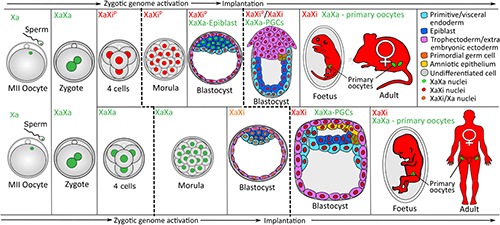
XCI during female mouse and human development. During mouse development, embryonic cells around the 4-cell stage inactivate the paternally inherited X chromosome. Cells of the primitive endoderm and trophectoderm in the preimplantation blastocyst keep this inactivation pattern while those in the epiblast reactivate the paternal Xi. Around implantation, an X chromosome is randomly inactivated within epiblast cells. Following specification of PGCs, these cells activate the Xi. Primary oocytes within the fetus and adult mouse do not contain an Xi while all somatic cells retain the Xi pattern of their epiblast precursor. During human development, random XCI is seen as a gradual process beginning in the early blastocyst and completing just prior to implantation, this pattern of inactivation is retained in all future somatic cells. Following specification of PGCs these cells reactivate the Xi which remains active in all future germ cells. Cells are colored by their lineage displayed in the upper right panel, primary oocytes (green) within the fetus and adults represent the primary oocytes contained in the ovaries. Relative timescales of mouse and human development are not accurately represented here. PGCs, primordial germ cells; Xa, active X chromosome; Xi, inactive X chromosome; Xip, inactive paternally inherited X chromosome. Reproduced and adapted with permission from Pasque and Plath (2015).

XCI and XCR are developmentally regulated processes (Figure 2). Several reporter mice have been created to enable live cell imaging of XCI and XCR *in vivo* (reviewed in Kobayashi, 2018). These reports have helped to define the dynamics of XCI and XCR (Hadjantonakis et al., 2001). In the early developing mouse embryo, XCI occurs in two waves. First, at embryonic day 3.5 (E3.5) the paternal X chromosome is inactivated and coated by *Xist* RNA and enriched for the repressive chromatin marks such as H3K27me3 and histone macroH2A1 (Costanzi et al., 2000; Mak et al., 2004; Okamoto et al., 2004). Imprinted XCI is maintained in most extra-embryonic tissues. However, between E3.5 and E4, XCI is followed by XCR in the ICM of the blastocyst (Mak et al., 2004; Okamoto et al., 2004; Borensztein et al., 2017a). Afterwards, at preimplantation stages, random XCI is initiated in the epiblast, which establishes X chromosome dosage compensation in the epiblast and in their somatic progeny cells. XCR also takes place during the formation of PGCs in mouse and human (Figure 2) (Mak et al., 2004; Okamoto et al., 2004; de Napoles et al., 2007; Sugimoto and Abe, 2007; Chuva de Sousa Lopes et al., 2008).

How genes on the X chromosome switch between transcriptionally active and inactive states during development has been the focus of numerous studies, mostly focusing on random XCI. During the initiation of XCI, *Xist* RNA induces chromosome-wide gene silencing (Penny et al., 1996; Monfort and Wutz, 2017). Relating to the kinetics of transcriptional silencing during XCI, several studies have shown that random and imprinted XCI are gradual, with genes inactivating early, mid, and late during XCI (Patrat et al., 2009; Marks et al., 2015; Żylicz et al., 2019). Different groups were able to identify the proteins bound to *Xist* RNA (Chu et al., 2015; McHugh et al., 2015; Minajigi et al., 2015), reviewed in Pinheiro and Heard (2017). A recent follow up study found that one of the first and necessary events for efficient XCI is HDAC3-mediated histone deacetylation (Żylicz et al., 2019). In addition, the recruitment of Polycomb by *Xist* RNA was found to be initiated by the non-canonical Polycomb Repressive Complex 1 (PRC1), which mediates ubiquitination of histone H2A, in turn leading to the recruitment of other PRC complexes and of PRC2 (Almeida et al., 2017). In agreement with this, Żylicz et al. (2019) reported that H2A ubiquitination is acquired in large intergenic regions followed by PRC2-associated H3K27me3 as one of the earliest XCI events. These marks are then spread into genetic regions only in the context of histone deacetylation and gene silencing (Żylicz et al., 2019). The specific genomic regions where *Xist* first binds, also known as *Xist* ‘entry’ sites, help to explain some, but not all of the gene silencing kinetics (Engreitz et al., 2013). Indeed, one possibility is that the chromatin landscape of the X chromosome prior to its inactivation instructs the folding of the chromosome, resulting in a specific pattern of *Xist* spreading and subsequent dynamics of gene silencing (Żylicz et al., 2019). In addition, the initiation of chromosome-wide gene silencing during XCI has been related to Polycomb entry sites (Pinter et al., 2012). Also, genetic features such as long interspersed nuclear elements (LINEs) and CTCF binding sites, have been implicated in efficient gene silencing during XCI (Pinter et al., 2012; Loda et al., 2017). By quantifying chromosome-wide gene silencing kinetics with allelic-resolution, using Precision nuclear Run-On sequencing (PRO-seq) and machine learning models, a recent study has found the linear distance and 3D interactions with the *Xist* locus to be predictive of gene silencing kinetics (Barros de Andrade et al., 2019), in agreement with previous studies (Marks et al., 2015; Borensztein et al., 2017a). However, proximity with LINE elements and gene density were associated with reduced silencing, in contrast with previous studies that reported *Xist* RNA spreading to gene-dense and LINE-poor regions (Engreitz et al., 2013; Simon et al., 2013). Recently, liquid–liquid phase separation has been hypothesized to be driven by *Xist* RNA and its binding partners in order to ensure effective XCI during its spreading and maintenance phases (Cerase et al., 2019). High-resolution microscopy has shown that the assemblies formed by *Xist* are similar in size and shape to the ones formed by other lncRNAs that drive liquid–liquid phase separation (Cerase et al., 2019). In addition, 54% of proteins that have been found to interact with *Xist* RNA are known to induce liquid–liquid phase separation in other biological contexts (Cerase et al., 2019). XCI also induces exclusion of RNA Polymerase II from the Xi domain, as well as late replication timing (Takagi, 1974; Chaumeil et al., 2006).

The plethora of chromatin changes taking place during XCI is accompanied by a shift toward XCI maintenance that is one of the most striking examples of stable gene silencing (Wutz and Jaenisch, 2000). In the maintenance phase of XCI, additional repressive modifications, such as DNA methylation, accumulation of macroH2A and hypoacetylation of histone H4, ‘lock in’ gene silencing on the Xi (Csankovszki et al., 2001). More recently, several screens have been reported, which aimed to further identify factors required to induce or maintain XCI (Minkovsky et al., 2015; Moindrot et al., 2015; Monfort et al., 2015; Sripathy et al., 2017; Carrette et al., 2018; Leko et al., 2018). The maintenance phase also includes weakening of self-interacting chromatin regions called topologically associating domains (TADs), and folding into two mega domains (reviewed in Jégu et al., 2017; Froberg et al., 2018; Galupa and Heard, 2018). Interestingly, deleting *Xist* or the architectural protein structural-maintenance-of-chromosomes hinge domain containing 1 (SmcHD1) on the Xi in somatic cells restores TAD formation but does not compromise XCI (Minajigi et al., 2015; Sakakibara et al., 2018; Gdula et al., 2019). Whether long-term stability of gene silencing is impaired in the absence of SmcHD1 is not clear, although this might be unlikely due to the abundance of other mechanisms in place to maintain XCI. In summary, the combined action of multiple pathways helps to stably maintain XCI.

## Timing of Gene Expression During X Reactivation in Mouse

Several lines of evidence have indicated that XCR proceeds gradually, with classes of genes reactivating with different timing; early, mid, or late during XCR (Borensztein et al., 2017a; Janiszewski et al., 2019). By using single-cell RNA-sequencing (scRNA-seq) of early mouse embryos, the timing of X-linked gene activation during the reversal of imprinted XCI in the ICM has been defined (Borensztein et al., 2017a). At E3.5, a subset of X-linked genes re-acquire bi-allelic expression. However, the remaining genes require an additional 12 h to reactivate. Importantly, the reactivation of the earliest genes precedes the downregulation of *Xist* RNA at E3.75 which suggests that *Xist* silencing is not required for all genes to reactivate (Williams et al., 2011; Borensztein et al., 2017a). The timing and gene-specific kinetics of XCR in the ICM do not mirror that of silencing during imprinted XCI (Borensztein et al., 2017a). Moreover, the dynamics of XCR do not correlate with the genomic location of *Xist* entry sites nor X-linked gene expression levels (Borensztein et al., 2017a, b). More studies are needed to decipher the additional factors influencing the kinetics of XCR in the ICM. For example, the *Xist* RNA ‘departure’ sites remain to be defined.

**FIGURE 3:**
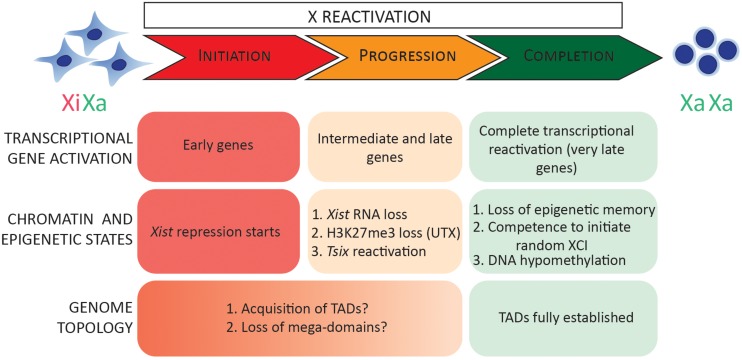
Stages of XCR. In this model, XCR can be divided into three phases: initiation, progression, and completion of XCR. During the early stages, early genes are transcriptionally activated and *Xist* repression is initiated. During XCR progression, *Xist* RNA, H3K27me3, and macroH2A are gradually lost from the Xi. *Tsix*, mid and late genes are transcriptionally reactivated. This process is coordinated by multiple chromatin pathways (such as UTX) as well as chromatin and transcriptional regulators (HDACs and TFs). In addition, TADs and mega-domains are reacquired throughout the initiation and progression phase of XCR. Finally, completion of XCR is characterized by a complete transcriptional reactivation of silenced genes, DNA hypomethylation, loss of epigenetic memory of XCI (random XCI or imprinted), and competence to initiate random XCI. Xa, active X chromosome; Xi, inactive X chromosome; TADs, topologically associating domains; XCI, X chromosome inactivation.

What is the timing of gene activation during the reversal of random XCI? Recently, allele-resolution RNA-sequencing (RNA-seq) of female mouse embryonic fibroblasts (MEFs) undergoing reprogramming to iPSCs has been reported (Janiszewski et al., 2019). A group of genes was found to reactivate ‘early,’ by day 8 of reprogramming, even before complete transcriptional activation of the pluripotency network took place. Another finding was that genes that reactivated early during iPSC reprogramming have a reduced genomic distance to escapee genes and tend to form ‘clusters’ (Janiszewski et al., 2019). Biallelic gene expression was detected for a subset of X-linked genes before complete *Xist* silencing, suggesting that during iPSC reprogramming, initiation of XCR might take place before *Xist* RNA is lost, similar to XCR in ICM. However, single-cell analyses are needed to confirm this finding. In analogy to XCI, where two phases have been reported (initiation and maintenance of XCI), XCR may operate via at least three phases: initiation, progression, and completion of XCR. Transcriptional activation of early genes and the start of *Xist* repression may represent the initiation stage of XCR (Figure 3).

The reversal of random XCI during iPSC reprogramming takes much longer than the erasure of gene silencing during the reversal of imprinted XCI in the ICM. During iPSC reprogramming, XCR takes 1 week, while it is completed in hours in the ICM (Borensztein et al., 2017a; Janiszewski et al., 2019). In addition, the genes that reactivate early in the ICM are different from those that reactivate early during iPSC reprogramming. The difference between the timing of reversal of imprinted XCI and random XCI might be explained by possible differences in the starting chromatin states, such as DNA methylation or macroH2A, and/or the presence of different *trans-*acting factors. Further work is needed to elucidate the starting chromatin states of the imprinted and random Xi.

Germ cell development also entails dramatic transcriptional and epigenetic reprogramming, including XCR, a physiological form of XCR (Sugimoto and Abe, 2007). Specification of germ cells initiates in the proximal epiblast, from where PGCs migrate through the hindgut toward genital ridges. In the future gonads, PGCs undergo meiosis and differentiate into sex-specific cells: eggs and sperm. Female germ cells initially downregulate *Xist* at E7.0 during the migration to genital ridges with a few genes re-acquiring bi-allelic expression (Chuva de Sousa Lopes et al., 2008). The rate of XCR significantly increases between E10.5 and E12, where most X-linked genes are expressed from both alleles, yet XCR is still incomplete at E14.5, meaning that XCR in PGCs is slower than XCR in the ICM (Sugimoto and Abe, 2007). XCR in PGCs is concordant with the loss of H3K27me3 enrichment on the X chromosome between E9.5 and E11.5 (de Napoles et al., 2007) and with two waves of DNA demethylation (Seisenberger et al., 2012). First, global DNA demethylation starts at E8, followed by locus-specific DNA demethylation in genes that were previously protected against demethylation. This second round of demethylation finishes around E13.5 (Hargan-Calvopina et al., 2016). Here, it was also found that conditional deletion of *Dnmt1* increased X-linked gene expression in PGCs (Hargan-Calvopina et al., 2016). In addition, PRDM14 has been shown to be important for XCR in PGCs by indirectly repressing *Xist* RNA via recruitment of PRC2 (Yamaji et al., 2008; Payer et al., 2013). Moreover, a very recent study showed that PRDM14 commonly regulates global and Xi enrichment of H3K27me3 in mouse PGCs (Mallol et al., 2019).

A recent study has revealed the timing of X-linked gene reactivation during mouse spermatogenesis (Ernst et al., 2019). Although fundamentally different from the reversal of random or imprinted XCI, male cells reactivate their only X chromosome during spermatogenesis after having undergone meiotic sex chromosome inactivation. The production of sperm cells is the outcome of a tightly orchestrated, unidirectional differentiation in which spermatogonial cells undergo a series of mitotic divisions until the spermatocyte stage, in which sex chromosomes are silent, followed by two meiotic divisions. Subsequently, the resulting spermatids differentiate to mature spermatozoa, during which X-linked genes are gradually activated (Ernst et al., 2019). Early reactivating genes included several members of the *Ssxb* gene family, which might function in post-meiotic XCR.

In summary, XCR is a gradual process that takes place multiple times during development and also during reprogramming to pluripotency. In most cases, the timing of X-linked gene activation seems to be gradual but the precise kinetics differ. In the next section, we discuss factors and mechanisms that might influence the timing of gene reactivation during XCR.

## Initiating Factors and Mechanisms of X Reactivation

*Xist* silencing is thought to be required but not sufficient for XCR in the mouse iPSC reprogramming system. Indeed, RNA-FISH experiments have revealed that *Xist* deletion during iPSC reprogramming is not sufficient to accelerate XCR (Pasque et al., 2014). However, these experiments only examined a few genes by RNA-FISH. Whether the timing of XCR is affected by *Xist* deletion needs to be reexamined using allele-specific RNA-seq. In addition, in the blastocyst, XCR is perturbed by mutations in either *Tsix* or *Prdm14* and during reprogramming, XCR is only affected by PRDM14 deficiency (Payer et al., 2013).

Ectopic *Xist* expression during iPSC reprogramming was found to delay XCR (Pasque et al., 2014). Therefore, *Xist* silencing is thought to be needed for XCR to take place. One exception may be the category of ‘early’ reactivated genes. One hypothesis to explain how *Xist* silencing takes place during XCR is that *Tsix*, the lncRNA transcribed in the opposite orientation to *Xist*, might help to repress *Xist* and mediate XCR. *In vivo*, *Tsix* deletion was reported to decrease the efficiency of XCR in the ICM as judged by H3K27me3 enrichment (Payer et al., 2013). However, *Tsix* deletion had little effect on XCR and did not prevent *Xist* repression during iPSC reprogramming (Payer et al., 2013; Pasque et al., 2014). The role of *Xist* and *Tsix* in XCR has been previously reviewed (Froberg et al., 2013; Pasque and Plath, 2015).

## The Influence of Chromatin on X Reactivation: Barriers and Mediators

In recent years, several groups have been able to identify inducers and barriers to XCR (Payer et al., 2013; Borensztein et al., 2017a; Janiszewski et al., 2019). In mouse, the paternal Xi in the ICM is enriched with chromatin modifications associated with gene repression such as H3K27me3 (Mak et al., 2004; Okamoto et al., 2004). Using allele-specific chromatin immunoprecipitation combined with deep sequencing (ChIP-seq) on early mouse embryos (Zheng et al., 2016), the Heard group was able to detect a clear and significant enrichment of H3K27me3 on the paternal Xi of ICM cells for late and very-late reactivated genes compared to early reactivated genes (Borensztein et al., 2017a). This suggested that H3K27me3 might oppose the transcriptional activation of late genes. Knock-out of *UTX*, a H3K27 demethylase, in mouse embryos, resulted in an increased biallelic expression of several late or very late and also early reactivated genes such as *Kif4, Rnf12, Pdha1, Abcd7*, and *Atrx* (Borensztein et al., 2017a). These experiments support the conclusion that H3K27me3 acts as a barrier to XCR in ICM at least for a subset of X-linked genes. Whether H3K27me3 opposes reversal of random XCI remains unclear. Interestingly, on the Xi of MEFs, H3K27me3 was enriched on all classes of genes (early, mid, late, and very late reactivated) without differences in H3K27me3 enrichment levels between early and late genes versus early genes (Janiszewski et al., 2019). Therefore, the starting chromatin state for the imprinted paternal Xi and randomly inactivated Xi may differently prime genes for reactivation in the ICM versus reprogramming to iPSCs.

Another chromatin protein that acts as a barrier to transcriptional activation is the histone variant macroH2A. Work from the Gurdon lab showed that removal of macroH2A from MEFs improves the efficiency of XCR following nuclear transfer (Pasque et al., 2011). Immunofluorescence studies have shown that deletion of *Xist* in somatic cells leads to the loss of enrichment of macroH2A and H3K27me3 on the Xi, probably by passive mechanisms during cell divisions (Csankovszki et al., 1999; Plath et al., 2004). Therefore, repressive histone modifications may be removed both by active and passive mechanisms following *Xist* silencing during XCR. In analogy to the stages of XCI, *Xist* RNA and chromatin marks associated with repression such as H3K27me3 and macroH2A are lost from the Xi during the progression stage of XCR (Figure 3). Meanwhile, *Tsix*, mid and late genes are transcriptionally activated.

Small molecule treatments during mouse iPSC reprogramming have confirmed that DNA methylation acts as a barrier to XCR (Pasque et al., 2014; Janiszewski et al., 2019). In addition, histone deacetylation has been identified as a barrier to XCR during reprogramming (Janiszewski et al., 2019). Thus, histone deacetylation is not only one of the first events during XCI, but it is also implicated in opposing XCR (Żylicz et al., 2019). Another study used *Xist* RNA live cell imaging and identified the histone acetyl transferase *Kat8* as necessary for reactivation of the Xi in the reversible phase of XCI (Ha et al., 2018). The precise mechanisms by which histone acetylation is regulated to mediate XCR remains to be explored. Reporter mice will help to enable live imaging of XCI and XCR and monitor the effects of histone acetylation (Kobayashi, 2018; Tran et al., 2018).

Moreover, cleavage under targets and release using nuclease (CUT&RUN) experiments during mouse spermatogenesis have identified a strong enrichment for H3K9me3, a chromatin modification associated with gene repression, in promoters of spermatid-specific genes expressed in spermatocytes (Ernst et al., 2019). These observations suggested H3K9me3 as a barrier for XCR, specifically for reactivation of those X-linked genes during spermatogenesis.

XCR entails dramatic changes also in chromatin topology (Stadhouders et al., 2018). Hi-C experiments during mouse iPSC reprogramming have revealed the chromosome-wide reacquisition of TADs on the X chromosome (Stadhouders et al., 2018). Are changes in topology sufficient for XCR? *Xist* deletion on the Xi of differentiated cells strengthens TADs but gene silencing is maintained suggesting that TAD formation is not sufficient for XCR (Minajigi et al., 2015; Carrette et al., 2018; Colognori et al., 2019).

Recently, chromatin organization and the TADs located in the X-inactivation center (*Xic*) have been implicated in the developmental timing of XCI (Nora et al., 2012; van Bemmel et al., 2019). The *Xic* is organized into two TADs, one containing *Xist* and the other containing *Tsix*. When *Xist* and *Tsix* were genomically inverted, their expression dynamics during XCI changed, with an upregulation of *Xist* in pluripotent stem cells (PSCs) and the expression of *Tsix* during PSC differentiation (van Bemmel et al., 2019). Does topology influence the timing of gene activation during XCR? A recent study found that the X-linked genes that reactivate early during iPSC reprogramming have a shorter genomic distance to escapee genes (Janiszewski et al., 2019). Since TADs are attenuated on the Xi but escapee genes maintain TAD-like structures on the Xi (Nora et al., 2012; Giorgetti et al., 2016), there might be a correlation between the presence of TADs or other 3D genome organization features at Xi-escapee genes and the early reactivated genes during XCR. Moreover, escapee genes have been shown to reside outside of the repressed compartment defined by *Xist* RNA (Chaumeil et al., 2006). *Xist* RNA has also been found to concentrate near escapee boundaries (Simon et al., 2013). It is possible that the chromatin organization of repressed genes on the Xi might influence the stability and timing of reversal of gene silencing during XCR. Silenced genes that are embedded in inner repressed compartments may be more resistant to transcriptional activation. Reacquisition of TADs during XCR might take place during the progression stage of XCR. During completion of XCR, there is complete transcriptional reactivation of silenced genes, loss of epigenetic memory of XCI (random XCI or imprinted), and competence to initiate random XCI is acquired (Figure 3).

The data obtained so far in different systems appears to point toward the repressive and synergistic role of multiple chromatin modifications, and possibly, chromatin organization in restricting XCR. In some cases, repressive modifications appear to be removed actively during XCR. In other cases, repressive chromatin modifications may be lost passively after *Xist* silencing. Additional unanswered questions regarding the influence of chromatin and topology in XCR include: what are the precise dynamics of chromatin organization during XCR? What are the dynamics of chromatin accessibility and modification changes during XCR? Do genes gradually relocate from the inner repressed compartment of the Xi to the outer compartment where they are activated? Does phase separation act as a barrier to XCR? Could it be that genes that are located in a more internalized space on the Xi are more difficult to reactivate due to topology acting as a barrier to XCR? The rapid development of improved techniques to investigate chromatin organization provides possible avenues to tackle these questions.

## Transcription Factors and X Reactivation in Mouse

A clear link exists between the pluripotency gene regulatory network and XCR. The presence of two active X chromosomes (Xas) is considered a robust hallmark of naïve pluripotency in mice and humans (Boroviak and Nichols, 2017). Several studies have established a link between the presence of a robust pluripotency network and *Xist* repression (Navarro et al., 2008; Deuve and Avner, 2011; Sousa et al., 2018). Indeed, the establishment of the naïve pluripotency network correlates well with the induction of *Xist* repression and with XCR in development and in reprogramming. For instance, in the mouse ICM, in PGCs and during the generation of iPSCs, NANOG expression marks cells that underwent XCR (Silva et al., 2009; Pasque et al., 2014). In addition, deletion of *Prdm14* has been reported to perturb XCR during iPSC derivation, as well as iPSC maintenance (Payer et al., 2013). Moreover, combined expression of *Prdm14* and *Klf2* synergistically induces reprogramming of epiblast stem cells to a naïve pluripotent state and XCR (Gillich et al., 2012).

Pluripotency factors influence *Xist* transcription in mESCs (Navarro et al., 2008; Sousa et al., 2018). For example, deletion of *Nanog* or *Oct4* in mESCs leads to an increase in *Xist* expression (Navarro et al., 2008). A recent study has shown that the destabilization of naïve pluripotency in mESCs by *Oct4* deletion leads to the upregulation of *Xist* transiently in males and on both X chromosomes in females. When *Oct4*, *Sox2*, *Klf4*, and *c-Myc* are expressed in female MEFs, *Xist* is not silenced immediately (Pasque et al., 2014), indicating that multiple (pluripotency) factors might influence the repression of *Xist*. These observations reinforce the idea that a robust pluripotency network inhibits *Xist* expression and helps to maintain two Xas. In mouse, the presence of two Xas in PSCs leads to an increase in the expression of several pluripotency genes and delays pluripotency exit, a phenomenon referred to as the ‘Schulz’ effect (Schulz et al., 2014; Song et al., 2019). *Xist* is also regulated by the *trans*-acting factor REX1 directly repressing *Xist* by binding to its promoter (Gontan et al., 2012).

Positive regulation of *Xist* is also known to be controlled by autosomal TFs, including Ying-Yang 1 (YY1) which is known to trigger the activity of *Xist* by binding to its promoter (Makhlouf et al., 2014). ChIP-seq previously revealed the strong binding of OCT4, SOX2, NANOG, and PRDM14 to the *Xist* intron 1 region in mESCs, which was proposed as the repressive mechanism linking pluripotency TFs to *Xist* repression (Navarro et al., 2008; Payer et al., 2013). However, a more recent study showed that the deletion of *Xist* intron 1 has little effect on XCI or XCR (Minkovsky et al., 2013). Therefore, the mechanisms linking pluripotency factors to *Xist* repression and X-linked gene activation need further study.

The *cis*-regulatory regions needed to repress *Xist* are located in the *Xic* (Lee et al., 1996). Several lncRNAs are encoded in the *Xic*, which have been implicated in regulating the expression of *Xist*, reviewed in van Bemmel et al. (2016). Among these lncRNAs, heterozygous deletion of *Jpx* in female mESCs was reported to decrease *Xist* expression from both alleles, suggesting that *Jpx* regulates *Xist* by acting in *trans* and *cis*, in a dose-dependent manner (Tian et al., 2010; Carmona et al., 2018). In addition, *Jpx* has been implicated in binding CTCF to evict it from the *Xist* promoter, allowing *Xist* expression (Sun et al., 2013). Transcription of *Ftx*, another *Xic*-linked lncRNA, has also been reported to activate *Xist* expression and proper *Xist* accumulation toward XCI, independently of the number of *Ftx* non-coding transcripts (Furlan et al., 2018). Altogether, this supports a strong link between a robust pluripotency network, *Xist* repression, chromatin structure and the presence of two Xas.

Recent studies suggested that binding of pluripotency TFs to promoters of X-linked genes might be important for XCR (Borensztein et al., 2017a; Janiszewski et al., 2019). For instance, X-linked genes that reactivate early in the ICM are enriched for C-MYC in mESCs (Borensztein et al., 2017a). Moreover, the genes that reactivate early during iPSC reprogramming are enriched for KLF4, SOX2 and C-MYC binding in mESCs (Janiszewski et al., 2019). PRDM14 has been identified as another TF that mediates XCR (Payer et al., 2013). Defining precise mechanisms of TF-mediated transcriptional activation could provide exciting insights into the understanding of XCR.

## X Reactivation in Human Cells

In humans, random XCI also takes place in preimplantation embryos (Figure 2) (van den Berg et al., 2009), although *XIST* RNA seems to be expressed differently than in mice (Okamoto et al., 2011). Unlike in the mouse, where only the Xi is coated by *Xist* RNA, in humans, both Xas in female and the sole X chromosome in male cells are coated with an *XIST* RNA cloud in human ICM cells (van den Berg et al., 2009; Okamoto et al., 2011). Thus, the initiation of XCI seems to differ between mice and humans. Another difference is that there is no imprinted XCI in humans hence no XCR occurs in the ICM. Early studies in human trophoblast cells reported that the paternal X chromosome is preferentially inactivated in this extra-embryonic tissue (Harrison, 1989; Goto et al., 1997). However, allele-resolution studies showed this process to be random (Moreira de Mello et al., 2010). Nevertheless, both X chromosomes are active in the human ICM (Okamoto et al., 2011; Petropoulos et al., 2016). Additionally, another lncRNA, *XACT*, is expressed and also forms an ‘RNA cloud’ on the human XaXa in early human embryos (Vallot et al., 2017; Casanova et al., 2019). Experiments in a mouse heterologous system suggested that *XACT* might be involved in the choice underlying random XCI (Vallot et al., 2017). However, to date, no *in vitro* experimental system enables the induction of random XCI starting from human undifferentiated PSCs (Theunissen et al., 2016; Sahakyan et al., 2017).

In recent years, progress has been made in studying the specification and epigenetic reprogramming of PGCs in the human embryo (Von Meyenn and Reik, 2015). In one study scRNA-seq was used to investigate XCR in human PGCs and XCR was found to occur as early as week 4 onwards during human embryo development (Guo et al., 2015). However, a more recent analysis also using scRNA-seq supports the idea that XCR takes place late during PGC development (Vértesy et al., 2018). It also reports that not all PGCs at 4–9 weeks have completed XCR. Instead, this study reveals the heterogeneity of XCR in PGCs. In addition, this study suggested that *XIST* expression is not predictive of XCR. Intriguingly, the expression of *XACT* was not detected. Increasing the number of single cells analyzed, and the sequencing coverage, as well as allelic resolution and RNA-FISH analyses, would help define the precise timing of XCR in the human germline and understand to what extent the XaXa state in the germline resembles the XaXa state in the ICM.

Different experimental strategies have been used to study human XCR *in vitro*, which is discussed in the next sections. Recently human female fibroblasts with an Xi fused with mESCs were used to study human XCR (Figure 1) (Cantone et al., 2016, 2017). Upon fusion, *XIST* RNA was rapidly lost, before cell division and the formation of proliferative hybrids (Cantone et al., 2016). Loss of H3K27me3 took place after 3 days and a number of genes became biallelically expressed, as revealed by RNA-FISH (Cantone et al., 2016). More recently, RNA-seq revealed that a subset of genes rapidly reactivated, while over 90% of X-linked genes were resistant to XCR in this system, supporting the idea that Xi silencing is very stable and opposed to transcriptional changes (Cantone et al., 2017). Factors that have been implicated in maintenance of XCI in humans include the activin A receptor type 1 (ACVR1), and 3-phosphoinositide-dependent protein kinase 1 (PDPK1) (Przanowski et al., 2018).

Another system to induce human XCR is the conversion of female fibroblasts or human primed (uneroded) PSCs to a naïve-like PSC state (Theunissen et al., 2016; Collier et al., 2017; Sahakyan et al., 2017; Vallot et al., 2017; Kilens et al., 2018). In this system, XCR is associated with *XIST* silencing followed by Xi reactivation and reacquisition of biallelic *XIST* expression in a subset of cells, as well as *XACT* reactivation and DNA demethylation (Vallot et al., 2015; Theunissen et al., 2016; Sahakyan et al., 2017; Vallot et al., 2017; Kilens et al., 2018). RNA-FISH and RNA-seq analyses revealed that XCR occurs after four passages in naïve media and is completed primarily during the late-stage of primed-to-naïve conversion (Collier et al., 2017; Sahakyan et al., 2017), in agreement with the notion that XCR is a hallmark of naïve pluripotency. Therefore, naïve PSCs mimic but do not fully recapitulate the embryo state of the X chromosomes. Moreover, female naïve human PSCs can undergo XCI when induced to differentiate (Sahakyan et al., 2017). Thus, differentiation of human naïve PSCs provides a system to study human XCI. Interestingly, XCR is followed by reacquisition of H3K27me3 enrichment on the *XIST* + Xas and dampening of X-linked gene expression (Sahakyan et al., 2017). However, human naïve PSCs converted from the primed state and induced to differentiate undergo non-random XCI (Sahakyan et al., 2017), such that it is the X chromosome that was previously inactive in the primed state that is selected for inactivation (Theunissen et al., 2016). Therefore, this indicates that the reactivated X chromosome retains some level of epigenetic memory in the naïve state. Future work investigating the properties of naïve PSCs will help in developing alternative human naïve PSC culture conditions able to fully erase epigenetic memory of XCI and thereby enable studying random XCI in humans, as well as early human development in general.

Partial to complete erosion of XCI can take place in human primed PSCs leading to heterogeneity of X chromosome status (Silva et al., 2008; Anguera et al., 2012; Mekhoubad et al., 2012; Barakat et al., 2015; Patel et al., 2017; Vallot et al., 2017). Erosion of XCI is characterized by loss of transcriptional silencing, loss of H3K27me3 enrichment, loss of DNA methylation and *XACT* reactivation (Patel et al., 2017; Vallot et al., 2017). A recent study by the Benvenisty lab has analyzed all available RNA-seq data for human female embryonic stem cell (ESC) and iPSC lines to determine X chromosome status (Bar et al., 2019). Most iPSC lines were found to retain an Xi, in agreement with a previous study (Tchieu et al., 2010), while most ESCs had eroded XCI (Bar et al., 2019). The cause of this difference is not clear but could come from differences in time in culture, culture condition or epigenetic memory of the cell of origin.

Recent work by the Anguera lab reported an unexpected upregulation of a number of X-linked genes in T cells from female patients with systemic lupus erythematosus, a disease with a higher incidence in females (Syrett et al., 2019). In addition, they also observed altered expression of *XIST* RNA interactome genes. One hypothesis proposed is that there may be perturbed XCI maintenance in T cells leading to XCR for selected genes. Allele-specific RNA-seq and RNA-FISH to detect biallelic X-linked gene expression could enable further testing of this exciting hypothesis.

Loss of the Xi or XCR has also been associated with cancer in humans and in mice. Loss of Xi, XCR and labile chromatin states have been reported in human breast cancer (Chaligné et al., 2015). Changes in X-linked gene expression have also been reported in other human tumor types (Sirchia et al., 2005; Pageau et al., 2007; Chaligné et al., 2015). In mouse, *Xist* loss has been linked to the development of aggressive blood cancers and to XCR (Yildirim et al., 2013). Therefore, *Xist* has been proposed as a potent suppressor of hematologic cancers. Whether changes in X-linked gene expression have a role in tumor growth remains unclear. A better understanding of the characteristic changes in XCI in tumors could be useful to resolve tumor heterogeneity and improve diagnostic and therapeutic strategies.

## Concluding Remarks

Collectively, recent studies that built upon the latest technological advances such as scRNA-seq, allele-resolution transcriptomics and epigenomics, as well as cellular reprogramming, have revealed that XCR is a gradual process. The timing of X-linked gene reactivation involves the combinatorial effects of multiple pathways including pluripotency and other TFs, chromatin modifications, histone writers and erasers as well as chromatin organization and lncRNAs. It will now be interesting to dissect how these layers interplay to control the kinetics of XCR. The enrichment of pluripotency TF binding on multiple X-linked genes suggests that these and other TFs could play a direct role in transcriptional activation.

TFs such as OCT4, NANOG, SOX2, PRDM14, and ESRRB might be directly involved in XCR and have also been implicated in *Xist* silencing (Navarro et al., 2008; Payer et al., 2013). Their binding to gene regulatory regions on the Xi, in combination of other TFs, could mediate the initiation, progression and completion of XCR. Histone modifications also appear to play a role in XCR by acting as barriers or mediators of transcriptional activation. So far, UTX and HDACs have been implicated in the removal of repressive and active chromatin marks to promote or oppose XCR, respectively. Recent studies also suggested a possible influence of chromatin topology on the maintenance and reversal of *Xist*-induced silencing. Moreover, novel factors involved in the exit from pluripotency and early lineage commitment could potentially act as mediators of XCI or barriers of XCR. For example, Polycomb targets which start to be active after epiblast specification (pluripotency exit), the ubiquitin-ligase *Rlim*, whose expression correlates with XCI (Barakat et al., 2011), and the DNA methyltransferase *Dnmt3a* and the transcriptional repressor *Zfp5J*, which negatively correlates with XCI (Mohammed et al., 2017), might play a role in XCR. In addition, the discovery of potential RNA interactors of *Xist* might shed light on XCR mechanisms. For this, techniques that enable to recognize interactions between two transcripts (e.g., *Xist* and potential RNA interactors) will need to be developed, similar to the one described in Quinodoz et al. (2018), where genome-wide detection of multiple, simultaneously occurring higher-order DNA interactions are detected. In this way, we might discover unknown principles by which XCI is reversed.

Altogether, the dynamics of XCR is likely the outcome of the combinatorial effect of lncRNAs, TFs, genome topology, and chromatin modifications. Understanding the reversal of gene silencing is important for at least three reasons. First, reactivation of silenced genes represents a potential therapy for diseases, such as Rett Syndrome. This strategy might extend to targeted reactivation of silenced tumor suppressor genes in cancer. Second, it is likely that revealing mechanisms of XCR will uncover gene regulation principles that are generally applicable, as it has already repeatedly been done in the past (Pasque et al., 2011; Nora et al., 2012). Third, reactivation of X-linked genes may underlie the emergence and/or progression of specific human disorders. Many intriguing questions are still waiting to be addressed and will open new avenues to link fundamental research with diseases and therapies.

## Author Contributions

IT, AJ, and VP wrote the manuscript with input from all the authors. JC and LV prepared the figures with the help of IT, AJ, and VP. All authors listed have approved the manuscript for publication.

## Conflict of Interest Statement

The authors declare that the research was conducted in the absence of any commercial or financial relationships that could be construed as a potential conflict of interest. The reviewer CR declared a past co-authorship with one of the authors VP to the handling Editor.
